# Effects of Lamotrigine and Topiramate on Glial Properties in an Astrocyte-Microglia Co-Culture Model of Inflammation

**DOI:** 10.1093/ijnp/pyab080

**Published:** 2021-11-16

**Authors:** Timo Jendrik Faustmann, Franco Corvace, Pedro M Faustmann, Fatme Seval Ismail

**Affiliations:** 1 Department of Psychiatry and Psychotherapy, Medical Faculty, Heinrich Heine University, Düsseldorf, Germany; 2 Department of Neuroanatomy and Molecular Brain Research, Ruhr University Bochum, Bochum, Germany; 3 International Graduate School of Neuroscience, Ruhr University Bochum, Bochum, Germany; 4 Department of Neurology, University Hospital Knappschaftskrankenhaus Bochum, Ruhr University Bochum, Bochum, Germany

**Keywords:** Astrocyte-microglia co-culture model, Connexin 43, inflammation, lamotrigine, topiramate

## Abstract

**Background:**

Astrocytes and microglia are involved in the pathophysiology of epilepsy and bipolar disorder with a link to inflammation. We aimed to investigate the effects of the antiepileptic and mood-stabilizing drugs lamotrigine (LTG) and topiramate (TPM) on glial viability, microglial activation, cytokine release, and expression of gap-junctional protein connexin 43 (Cx43) in different set-ups of an in vitro astrocyte-microglia co-culture model of inflammation.

**Methods:**

Primary rat co-cultures of astrocytes containing 5% (M5, representing “physiological” conditions) or 30% (M30, representing “pathological, inflammatory” conditions) of microglia were treated with different concentrations of LTG and TPM for 24 hours. An 3-(4,5-dimethylthiazol-2-yl)-2,5-diphenyltetrazolium bromide (MTT) assay was performed to measure the glial cell viability. The microglial activation state was analyzed by immunocytochemistry. The pro-inflammatory tumor necrosis factor-α (TNF-α) and anti-inflammatory transforming growth factor-ß1 (TGF-ß1) cytokine levels were measured by enzyme-linked immunosorbent assay. The astroglial Cx43 expression was quantified by western blot.

**Results:**

A significant reduction of the glial cell viability after incubation with LTG or TPM was observed in a concentration-dependent manner under all conditions. LTG caused no significant alterations of the microglial phenotypes. Under pathological conditions, TPM led to a significant concentration-dependent reduction of microglial activation. This correlated with increased astroglial Cx43 expression. TNF-α levels were not affected by LTG and TPM. Treatment with higher concentrations of LTG, but not with TPM, led to a significant increase in TGF-ß1 levels in M5 and M30 co-cultures.

**Conclusions:**

Despite the possible glial toxicity of LTG and TPM, both drugs reduced inflammatory activity, suggesting potential positive effects on the neuroinflammatory components of the pathogenesis of epilepsy and bipolar disorder.

**Significance Statement**

Apart from neurons, glial cells and their role in inflammation have become of increasing interest for understanding neuropsychiatric diseases such as epilepsy and bipolar disorder. Lamotrigine (LTG) and topiramate (TPM) are important antiepileptic drugs with mood-stabilizing effects, which are frequently used in clinical treatment. Previous studies have indicated their effects on neurons, glial cells, and inflammation. The present study offers new findings on LTG and TPM effects in physiological and pathological set-ups of an astrocyte-microglia co-culture model of inflammation. Treatment with LTG and TPM reduced the glial cell viability, indicating possible toxic effects. On the other hand, both drugs were suitable for reducing inflammatory activity in glial co-cultures, suggesting potential positive effects on the neuroinflammatory components of the pathogenesis of epilepsy and bipolar disorder. The astrocyte-microglia co-culture model made possible to study the endogenous inflammatory reaction and cytokine expression under drugs in a differentiated manner.

## Introduction

### Epilepsy, Glia-Mediated Inflammation, and Antiepileptic Drugs (AEDs)

Epilepsy is one of the most common and most disabling neurological disorders, affecting all age groups and requiring drug treatment. There is increasing evidence that glial cells comprising astrocytes and microglia play a role in the pathophysiology of epilepsy (Patel et al., 2019). Astrocytes, the main glia cell population in the CNS, are involved in the support of neuronal network as well as maintenance of transmitter and ion homeostasis. Further, they are part of the tripartite synapse composed of pre- and postsynaptic neurons and perisynaptic astrocytic processes as a functional unit (Araque et al., 1999; Möller et al., 2007). Dysfunction of specific astrocytic membrane channel proteins, such as potassium channel K_ir_4.1, water channel aquaporin 4, glutamate transporters, or gap junction (GJ) protein connexin 43 (Cx43), which is the main GJ protein in astrocytes, has been reported to be involved in the pathophysiology of epilepsy (Patel et al., 2019).

Microglia are the main immune cells of the CNS. In the healthy brain, they are found in a resting ramified type (RRT) form ranging from 5% to 20% of the glial cell population (Faustmann et al., 2003). Microglial activation under pathological conditions comprises proliferation of microglia, change of the morphological phenotype from the resting ramified type (RRT) to the activated, rounded phagocytic type (RPT), expression of immune molecules, and release of inflammatory mediators (Gehrmann et al., 1995). The intermediate type of microglia, which is characterized by short cell processes, is the phenotypic transition from RRT to RPT. Clinical and experimental evidence confirms that epileptic activity is accompanied by molecular inflammatory mediators (e.g., interleukin [IL]-1β, tumor necrosis factor [TNF]-α, and IL-6) produced by glia, neurons, endothelial cells of the blood–brain barrier, and peripheral immune cells as well as by cellular mechanisms including, for example, reactive astrocytosis and activated microglia (Vezzani and Granata 2005; Vezzani et al., 2011). As a step before establishing new strategies or targets, it seems important to have more knowledge about the anti-/pro-inflammatory effects of currently available AEDs.

In previous reports, pro-inflammatory as well as anti-inflammatory effects of the AEDs levetiracetam (LEV), valproic acid (VPA), gabapentin, phenytoin, and carbamazepine (CBZ) have been shown to be glia mediated (Stienen et al., 2011; Dambach et al., 2014). As 1 parameter, microglia in-/activation state was triggered by the AEDs LEV, VPA, phenytoin, and CBZ in astrocyte-microglia in vitro co-cultures (Stienen et al., 2011; Dambach et al., 2014). Strong microglial activation was induced by VPA, whereas CBZ significantly reduced the amount of activated microglial cells (Dambach et al., 2014). In another in vitro study, metabolic effects of GBP, CBZ, lamotrigine (LTG), topiramate (TPM), oxcarbazepine, tiagabine, and LEV on primary astrocytes were demonstrated (Pavone and Cardile 2003).

LTG [3,-5-diamino-6-(2,3-dichlorophenyl-1,2–3-triazine)] and TPM [2,3:4,5-di-O-isopropylidene-β-D-fructopyranose sulfamate] are well-established anticonvulsants. LTG has been also approved for the treatment of bipolar disorder (BD). TPM has not been approved for the treatment of mood disorders or other psychiatric disorders by the European Medicines Agency or the US Food and Drug Administration. The antiepileptic properties of LTG include inhibition of voltage-sensitive sodium channels in neuronal membrane, inhibition of release of the excitatory neurotransmitters glutamate and aspartate, and blockade of calcium channels (Miranda et al., 2019). The antiepileptic efficacy of TPM is based on multiple mechanisms of action, including voltage-sensitive sodium channel blockade, calcium channel inhibition, increase of potassium conductance, GABA-mediated chloride current increment, glutamate-mediated neurotransmission inhibition, and carbonic anhydrase isoenzyme inhibition (Guerrini and Parmeggiani, 2006).

### BD, Glia-Mediated Inflammation, and Mood-Stabilizing Drugs

BD belongs to the field of mood disorders in clinical psychiatry. Symptoms vary between mania, hypomania, or episodes of depression, but mixed episodes can also occur. The pathogenesis of BD is poorly understood and experimental models are rare (Miranda et al., 2019). A link between BD, inflammation, and cytokine levels is known and points to the cytokine-hypothesis of mood disorders (Fiedorowicz et al., 2015; van den Ameele et al., 2016). Similar to epilepsy, the role of glia cells (astrocytes, microglia) in the pathophysiology of BD is strongly discussed, but exact mechanisms are not understood (Pinto et al., 2018). Post-mortem studies showed an increased lateralization of microglia density in the right hemisphere of BD patients (Petrasch-Parwez et al., 2020). Further, a high lifetime prevalence of BD in patients with inflammatory CNS disease such as multiple sclerosis was reported (Joseph et al., 2021).

There is evidence that psychotropic drugs such as venlafaxine have immunomodulatory functions, suggesting a crosslink between glial cells, inflammation, and psychiatric disorders (Vollmar et al., 2008, 2009). The AEDs LTG and TPM revealed different effects in the treatment of BD. LTG has more effect on bipolar depression than mania (Calabrese et al., 2003; Prica et al., 2008). The mood-stabilizing effect of LTG in bipolar depression might be based on involvement of the monoaminergic (serotonergic, noradrenergic, and dopaminergic) and glutamatergic systems (involving NMDA- and AMPA-receptors), and improvement of mania could refer to regulatory effects on AMPA-receptors (Rapoport et al., 2009; Miranda et al., 2019). TPM has not been approved for the treatment of BD and seems to be less effective (Kushner et al., 2006; Pigott et al., 2016). Previous findings about effects of AEDs on cytokine levels in BD are limited (van den Ameele et al., 2016). In murine models of inflammation, LTG significantly inhibited IL-1β, IL-6, and TNF-α secretion in vivo and in vitro, revealing possible immunomodulatory properties of LTG in epilepsy and BD (Abu-Rish et al., 2018).

### Astrocyte-Microglia Co-Culture Model of Inflammation

Faustmann et al., 2003 developed an in vitro astrocyte-microglia co-culture model of inflammation, which is an established cell-culture model of glial cells, demonstrating a functional relationship between microglial activation and coupling efficiency in the astroglial network. The M5 and M30 co-cultures present depending on microglia content and activation status experimental conditions mimicking normal (healthy) or diseased brain. The M5 co-cultures contained mainly resting ramified microglia cells, whereas activated microglia were found in M30 co-cultures. Incubation of M5 co-cultures with the pro-inflammatory cytokines TNF-α, IL-1β, IL-6, and interferon (IFN)-ү led to microglial activation (Hinkerohe et al., 2005). Otherwise, addition of the anti-inflammatory cytokine transforming growth factor (TGF)-ß1 to inflammatory M30 co-cultures caused a reduction of microglial activation and reconstitution of functional coupling (Hinkerohe et al., 2005). Furthermore, IFN-β prevented the effects of the pro-inflammatory cytokines TNF-α, IL-1β, and IFN-ү in M5 co-cultures (Hinkerohe et al., 2005). The effects of different drugs, for example, AEDs and immunomodulatory and psychotropic drugs, have already been tested in our co-culture model (Haghikia et al., 2008; Vollmar et al., 2008; Hinkerohe et al., 2011; Stienen et al., 2011; Dambach et al., 2014). In summary, this unique in vitro model is suitable for investigation of the endogenous inflammatory reaction and cytokine expression under drugs in a differentiated manner.

### Aim of the Study

In this study, we aimed to investigate the effects of the antiepileptic and mood-stabilizing drugs LTG and TPM on glial viability, microglial activation and morphology, cytokine release, and expression of GJ protein Cx43 in different set-ups of an in vitro astrocyte-microglia co-culture model of inflammation.

## METHODS

### Cell Culture

As previously described by Faustmann et al. (2003), astrocyte-microglia co-cultures were prepared using brains of postnatal Wistar rats (postnatal day 0–2, P0–P2). Experiments were approved by the local authorities in Bochum, Germany, and performed according to the German Animal Welfare Act and the ethical standards of Ruhr University Bochum. Animals had free access to food and water and were kept under standard laboratory conditions. According to the German Animal Welfare Act, the P0–P2 rats were decapitated without sedation. Cerebellum, meninges, and choroid plexus were removed, and the brains were kept in ice-cold phosphate buffered saline (PBS) (containing 1.38 M NaCl, 27 mM KCl, 81 mM NaH_2_PO_4_, 14.7 mM K_2_H_2_PO_4_ (J.T. Baker, Deventer, the Netherlands). After treatment with 0.1% trypsin (PAA Laboratories, Pasching, Austria) for 30 minutes at 37°C, they were centrifuged at 500×g for 12 minutes and the supernatant removed. The pellet was resuspended in 5 mL of DNase I solution (Serva Electrophoresis, Heidelberg, Germany) (100 µL/mL with Dulbecco’s minimal essential medium; Invitrogen, Karlsruhe, Germany) for 5 minutes at room temperature. Centrifugation at 200×g for 5 minutes and washing steps (washing medium containing 10% fetal calf serum [Biochrom AG, Berlin, Germany], 1% penicillin/streptomycin solution [PAA Laboratories, Linz, Austria]) were performed. Following this, the pellet was filtered through a 60-μm nylon mesh. One brain per plastic tissue-culture flask was cultured in 7% CO_2_ at 37°C in astrocyte culture medium (containing 10% fetal calf serum, 1% non-essential amino acids, 1% glutamine, 1% penicillin/streptomycin solution) (PAA Laboratories). The cultures reached approximately 100% confluency after 5 days. By manual shaking the flasks, adherent microglial cells and oligodendroglia on the astroglial surface were removed. A variation between 5% and 10% and between 30% and 40% of microglial cells was found in the co-cultures depending on the extent of shaking. The amount of microglial cells was determined by counting the cells after fixation and staining.

### Treatment of Cultures

Based on previous studies with LTG and TPM (Pavone and Cardile 2003; Abu-Rish et al., 2018) and corresponding to measured serum concentrations of patients with epilepsy (St Louis, 2009), the primary rat glial co-cultures of astrocytes containing 5%–10% (M5, representing “physiological” conditions) or 30%–40% (M30, representing “pathological” conditions) of microglia were incubated with different concentrations of LTG or TPM (5, 10, 25, and 50 µg/mL) (Sigma-Aldrich, Steinheim, Germany) for 24 hours in 7% CO_2_ at 37°C. The drugs were dissolved in dimethyl sulfoxide and diluted in culture medium. The control cell co-cultures were not exposed to the vehicle dimethyl sulfoxide used to dissolve the drugs. The controls were untreated with any substance/vehicle.

Because measured serum, cerebrospinal fluid, and brain tissue concentrations of AEDs showed intraindividual and interindividual variation in previous investigations (Rambeck et al., 2006), we attempted to detect concentration-dependent effects.

### MTT Assay

The viability, proliferation, and cytotoxicity of cells were measured using a 3-(4,5-dimethylthiazol-2-yl)-2,5-diphenyltetrazolium bromide) (MTT) assay (Roche Applied Sciences, Mannheim, Germany). Co-cultures were transferred from tissue-culture flask onto poly-L-lysine–coated glass cover slips at 10 000 cells per well in 94-well plates in 7% CO_2_ at 37°C and were cultured until they were confluent. Co-cultures were treated with LTG or TPM as described above. Incubation with 10 µL 3-(4,5-dimethylthiazol-2-yl)-2,5-diphenyltetrazolium bromide) (MTT) reagent for 4 hours in 7% CO_2_ at 37°C was performed. Following this, 100 µL of solubilization solution was applied to the co-cultures, and the samples were incubated overnight. Using the Bio-Rad Microplate Reader (München, Germany), the cell viability in the wells was measured the next day at a wavelength of 550 nm.

### Immunocytochemistry

**Figure 1.**
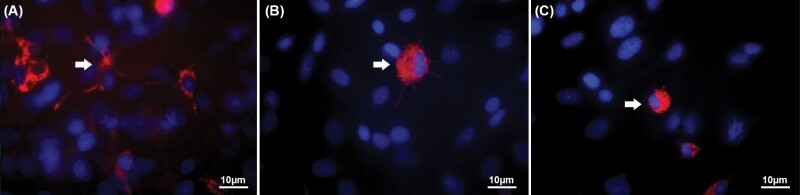
Immunohistochemistry of microglia morphology (white arrows) at a magnification of ×630. ED1 staining (red) allowed the classification of microglia as resting ramified (A), intermediate (B), and activated rounded phagocytic (C) phenotype under treatment with lamotrigine or topiramate. The representative images used in the figure were captured after incubation of M5 astrocyte-microglia co-cultures with lamotrigine at a concentration of 50 µg/mL. The cells were counterstained with 4,6-diamidino-2-phenyl-indol (DAPI) to visualize the nuclei (blue) of the total glial cell number. Scale = 10 µm.

The microglial phenotypes were analyzed by immunocytochemistry. As described by Faustmann et al. (2003), microglia were labeled by using multiple antibody markers such as ED1, OX-42, and Isolectin B4 during the development of the astrocyte-microglia co-culture model. The ED1 antibody showed the most intense immunoreactivity of the tested microglial/macrophage markers and allowed differentiation of microglial subtypes (Figure 1A–C). Therefore, we used the ED1 antibody in further experiments with the astrocyte-microglia co-culture model to visualize the microglial phenotypes/morphology with regard to activation status. The astrocyte-microglia co-cultures were placed on poly-L-lysine–coated glass cover slips at 70 000 cells per well in 24-well plates and incubated with LTG or TPM as described above. Fixation of cover slips with 70% ethanol for 10 minutes and incubation in PBS-blocking solution containing 1% bovine serum albumin (1% BSA) (PAA Laboratories) were performed. After treatment with mouse anti-ED1 (1:250) (Serotec, Düsseldorf, Germany) (diluted in 1% BSA in PBS), the cover slips were incubated overnight at 4°C. In the next step, the wells were incubated with goat anti-mouse immunoglobulin G conjugates (Alexa fluor 568) (1:500) (diluted in 1% BSA, 10% horse serum in PBS) (Invitrogen) for 1 hour. For quantification of cell numbers, immunocytochemically labeled cells were counterstained with 4,6-diamidino-2-phenyl-indol (DAPI; 1:2500) (Invitrogen). By comparison of the number of ED1-stained microglia with the total number of DAPI-labeled cells, the ratio of microglia to astrocytes was detected. Evaluation of microglia morphology was carried out in a minimum of 3 different visual fields on each cover slip at a primary magnification of ×630. ED1 staining led to the classification of microglia as ramified, intermediate, and activated rounded phagocytic phenotype (Faustmann et al., 2003) (Figure 1A–C).

### Enzyme-Linked Immunosorbent Assay (ELISA)

ELISA was performed using 2 kits for measurement of supernatant concentrations of TGF-ß1 and TNF-α according to the manufacturer’s instructions (Promega, Madison, WI, USA; R&D Systems, Minneapolis, MN, USA). After dilution of the primary antibody (anti-rat TNF-α or anti-rat TGF-ß1), 100 μL was added to each well of a microtiter plate (96-well plate). Following this, the microtiter plate was covered with parafilm and incubated overnight at room temperature. The next day, the microtiter plate was washed 3 times with wash buffer and then blocked with 300 μL Reagent Diluent for 1 hour at room temperature. After washing again, 100 μL of the standard solution or samples was added to each well, sealed with parafilm, incubated for 2 hours at room temperature, and then washed again. In the next step, 100 μL of the detection antibody was added to the wells of the microtiter plate, which were sealed, incubated for 2 hours at room temperature, and then washed. After addition of 100 μL streptavidin horseradish peroxidase, incubation in the dark for 20 minutes was performed with a washing step. Next, 100 μL of substrate solution was added. After 20 minutes incubation, 50 μL of stop solution (2N H_2_SO_4_) was applied. The optical density was measured within 30 minutes by using a Bio-Rad Microplate Reader (Bio-Rad 550, Hercules, CA, USA) at 450 nm.

### Immunoblot (Western-Blot) Analysis

Quantification of all forms of Cx43 (panCx43) expression by immunoblot analysis was performed according to the protocol. On each dish, 300 000 cells were seeded. With reaching confluency, the cells were treated with LTG or TPM as described above. After washing with PBS and lysis with 200 μL Laemmli 1 × buffer and 4 μL protease inhibitor cocktail, the cells were detached from the culture dishes using a silicone scraper, and the lysates were kept on ice. Using the Bradford assay (Bio-Rad Bradford Protein Assay, München, Germany), the protein concentrations were measured based on the protocol (Bradford, 1976). Loading of 10 μg solution onto 10% or 15% sodium dodecyl sulfate gel (AppliChem, Darmstadt, Germany) was carried out. Electrophoresis was done at 100 V for 20 minutes followed by 150 V. The gels were transferred to nitrocellulose membrane and blocked with Odyssey blocking buffer (LI-COR Bioscience, Bad Homburg, Germany) for 1 hour. Next, membranes were incubated overnight with anti-β-actin (1:10 000) (Sigma, St. Louis, MO, USA) or anti-Cx43 (1:5000) (Invitrogen) antibodies (diluted in 0.5% blocking buffer) at 4°C. Membranes were washed with 0.1% Tween20 (AppliChem) in PBS for 3 × 15 minutes and were treated with secondary anti-β-actin peroxidase goat anti-mouse (1:20 000) and peroxidase goat anti-rabbit (1:10 000) fluorescent antibodies (Sigma) (diluted in 0.5% blocking buffer) for 1 hour. After washing again with Tween20 in PBS, bands were visualized using the Odyssey Infrared Imaging System (LI-COR Bioscience, Germany). ImageStudio Lite V5.2 software from LI-COR was used for subsequent quantification of the bands. Cx43 was quantified using Microsoft Excel in ratio to the β-actin band.

### Data Analyses and Statistics

GraphPad Prism version 7.0 for Windows (GraphPad Software, San Diego, CA, USA) was used for statistical analyses and graphs. The Kolmogorov-Smirnov tests and D’Agostino-Pearson omnibus tests were performed for analyzing normality of data distribution. When normality was given, parametric tests were applied. Comparisons between more than 2 groups with normal distribution were analyzed using 1-way ANOVA followed by Kruskal-Wallis test or Bonferroni post hoc comparison test. Differences were considered to be statistically significant at *P* < .05. The results were reported as mean ± SEM.

## RESULTS

### Effects of LTG and TPM on Glial Cell Viability

**Figure 2.**
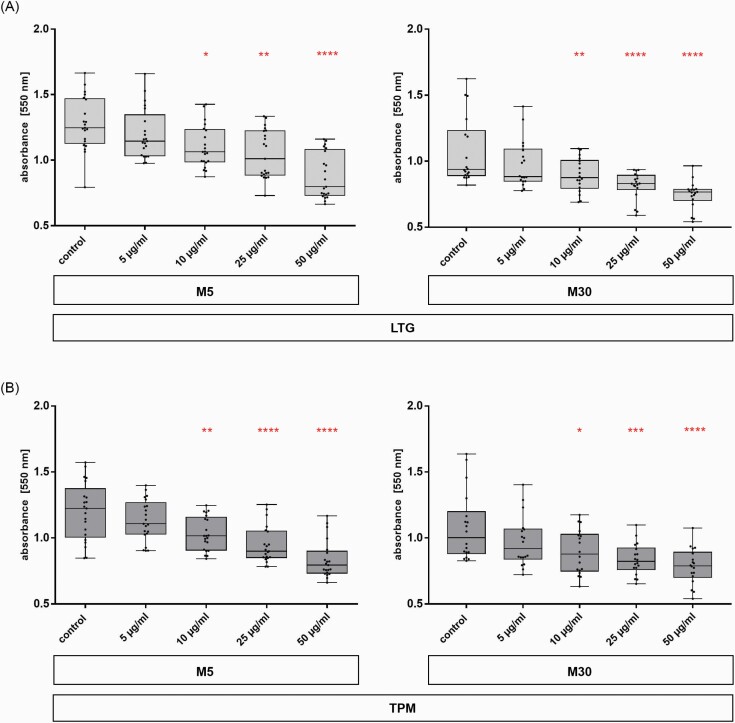
Glial viability detected by 3-(4,5-dimethylthiazol-2-yl)-2,5-diphenyltetrazolium bromide (MTT) assay in M5 and M30 co-cultures after concentration-dependent incubation (5–50 µg/mL) with lamotrigine (LTG) (A) and topiramate (TPM) (B) for 24 hours. The viability of M5 (n = 21 independent experiments) as well as M30 co-cultures (n = 18 independent experiments) was concentration-dependently reduced after incubation with LTG and TPM for 24 hours (A) (cell viability in M5 after LTG: control 1.28 ± 0.04; 5 µg/mL 1.2 ± 0.04; 10 µg/mL 1.1 ± 0.04; 25 µg/mL 1.05 ± 0.04; 50 µg/mL 0.88 ± 0.04; cell viability in M30 after LTG: control 1.071 ± 0.06; 5 µg/mL 0.98 ± 0.04; 10 µg/mL 0.89 ± 0.03; 25 µg/mL 0.81 ± 0.02; 50 µg/mL 0.74 ± 0.03); (B) (cell viability in M5 after TPM: control 1.2 ± 0.05; 5 µg/mL 1.13 ± 0.03; 10 µg/mL 1.03 ± 0.03; 25 µg/mL 0.94 ± 0.03; 50 µg/mL 0.8 ± 0.03; cell viability in M30 after TPM: control 1.08 ± 0.06; 5µg/mL 0.97 ± 0.04; 10µg/mL 0.9 ± 0.04; 25µg/mL 0.8 ± 0.03; 50µg/mL 0.78 ± 0.03). Comparisons between means were analyzed using D’Agostino-Pearson normality test and 1-way ANOVA followed by Bonferroni post hoc comparison test. Differences were considered significant at *P < .05; **P < .01; ***P < .001; ****P ≤ .0001.

Incubation with LTG for 24 hours significantly reduced the glial cell viability of the M5 co-cultures, representing “physiological” conditions, at concentrations of 10, 25, and 50 µg/mL (n = 21; *P < .05; **P < .01; ****P ≤ .0001) (Figure 2A). In M30 co-cultures, representing “pathological” conditions, a significantly reduced viability was observed after incubation with LTG at concentrations of 10, 25, and 50 µg/mL (n = 18; **P < .01; ****P ≤ .0001) (Figure 2A).

Incubation of the M5 co-cultures with increasing concentrations of TPM for 24 hours significantly reduced the viability of the glial cells (10, 25, and 50 µg/mL) (n = 21; **P < .01; ****P ≤ .0001) (Figure 2B). The M30 co-cultures also showed a significant decreased viability at concentrations of 10, 25, and 50 µg/mL TPM (n = 18; *P < .05; ***P < .001; ****P ≤ .0001) (Figure 2B).

### Influence of LTG and TPM on the Microglial Activation Under Physiological and Pathological Conditions

**Figure 3.**
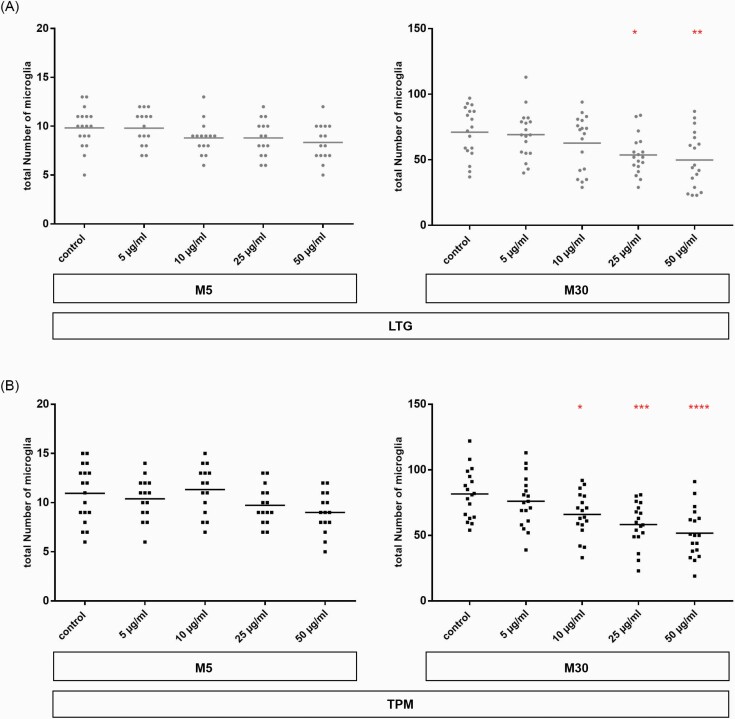
A total number of microglia by immunocytochemistry in M5 and M30 co-cultures after concentration-dependent incubation (5–50 µg/mL) with lamotrigine (LTG) (A) and topiramate (TPM) (B) for 24 hours. In M5 co-cultures, incubation with LTG or TPM for 24 hours did not change the total number of microglia (n = 18). In M30 co-cultures, a significant reduction of the total number of microglia was observed after incubation with LTG at concentrations of 25 and 50 µg/mL and after incubation with TPM at concentrations of 10, 25, and 50 µg/mL (A) (microglia number in M5 after LTG: control 9.8 ± 0.5; 5 µg/mL 9.8 ± 0.5; 10 µg/mL 8.8 ± 0.4; 25 µg/mL 9.8 ± 0.5; 50 µg/mL 8.3 ± 0.5; microglia number in M30 after LTG: control 71 ± 4.5; 5 µg/mL 69 ± 4.3; 10 µg/mL 63 ± 5; 25 µg/mL 54 ± 3.6; 50 µg/mL 50 ± 5); (B) (microglia number in M5 after TPM: control 10 ± 0.7; 5 µg/mL 10 ± 0.5; 10 µg/mL 11 ± 0.6; 25 µg/mL 9.7 ± 0.5; 50 µg/mL 9 ± 0.5; microglia number in M30 after TPM: control 81 ± 4.4; 5 µg/mL 76 ± 4.6; 10 µg/mL 66 ± 3.9; 25 µg/mL 58 ± 3.9; 50 µg/mL 52 ± 4.4). Comparisons between means were analyzed using D’Agostino-Pearson normality test and 1-way ANOVA followed by Bonferroni post hoc comparison test. Differences were considered significant at *P < .05; **P < .01; ***P < .001; ****P ≤ .0001.

Incubation with LTG or TPM for 24 hours did not influence the total number of microglia by immunocytochemistry under physiological conditions (M5 co-cultures) (n = 18) (Figure 3A and B). In M30 co-cultures, a significant reduction of the total number of microglia was observed after incubation with LTG at a concentration of 25 and 50 µg/mL (n = 18; *P < .05; **P < .01) (Figure 3A). Similarly, incubation with TPM at concentrations of 10, 25, and 50 µg/mL led to significant decrease of the microglia number under pathological conditions (n = 18; *P < .05; ***P < .001; ****P ≤ .0001) (Figure 3B). The number of astrocytes decreased after incubation with increasing concentrations of LTG more in M30 than in M5 co-cultures. After incubation of M5 and M30 co-cultures with TPM, there was no relevant change in the astrocyte number (data not shown).

**Figure 4.**
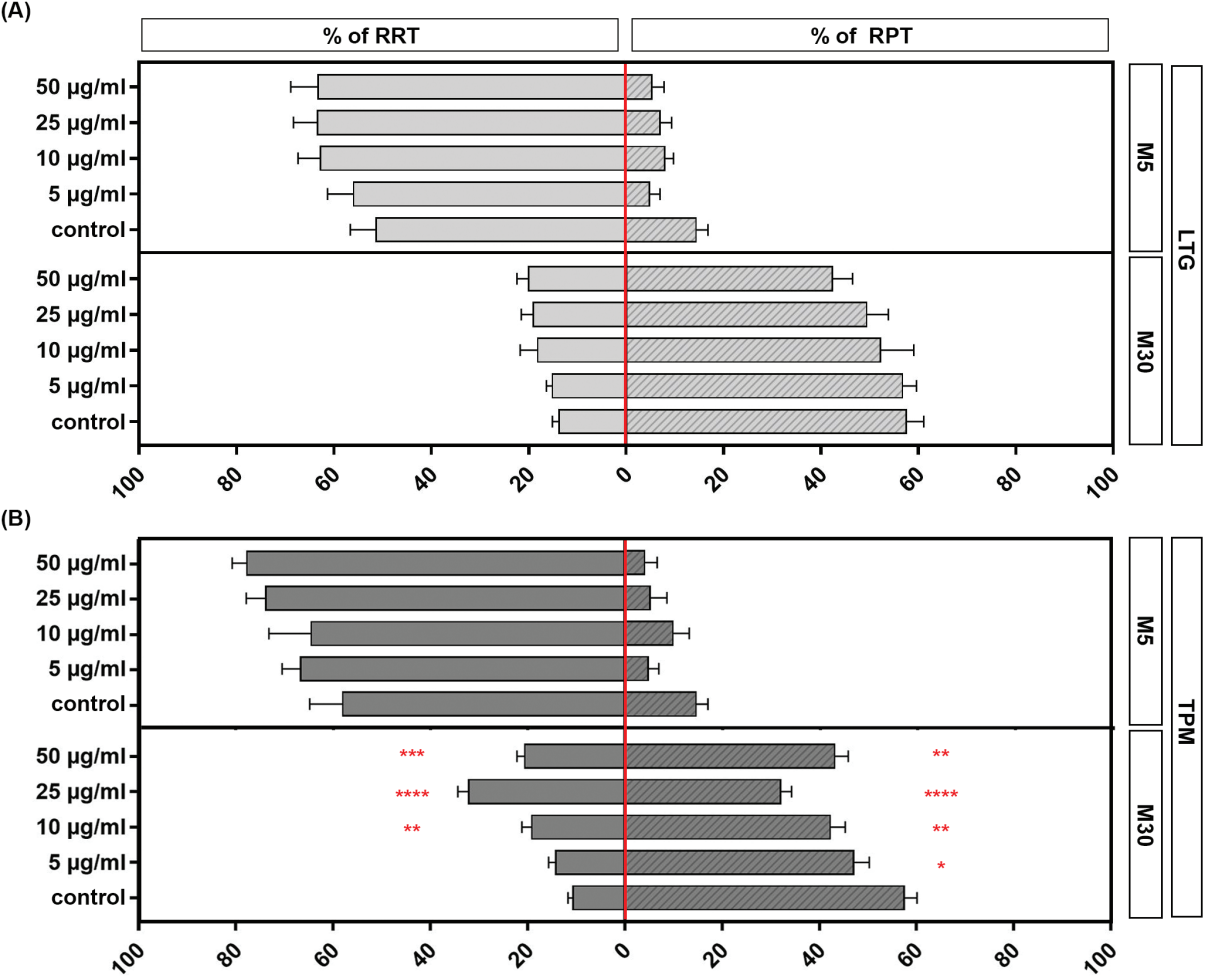
Immunocytochemical analyses of microglial phenotypes after concentration-dependent incubation [5–50 µg/mL] with lamotrigine (LTG) (A) and topiramate (TPM) (B) for 24 hours under physiological (M5) and pathological conditions (M30). Incubation with all tested concentrations of LTG for 24 hours caused no significant alterations of the microglial phenotypes in M5 as well as M30 co-cultures (n = 18) (A) (LTG effects on M5 resting ramified type of microglia (RRT): control 51.3% ± 5.2; 5 µg/mL 55.9% ± 5.3; 10 µg/mL 62.7% ± 4.5; 25 µg/mL 63% ± 4.9; 50 µg/mL 63% ± 5.6; LTG effects on M5 rounded phagocytic type of microglia (RPT): control 14.6% ± 2.4; 5 µg/mL 4.9% ± 2.2; 10 µg/mL 8.1% ± 1.8; 25 µg/mL 7.1% ± 2.4; 50 µg/mL 5.4% ± 2.54; LTG effects on M30 RRT: control 13.8% ± 1.4; 5 µg/mL 15% ± 1.2; 10 µg/mL 18% ± 3.6; 25 µg/mL 19% ± 2.4; 50 µg/mL 20 ± 2.4; LTG effects on M30 RPT: control 57.5% ± 3.6; 5 µg/mL 56.7% ± 2.9; 10 µg/mL 52.2% ± 6.8; 25 µg/mL 49.4% ± 4.4; 50 µg/mL 42.4% ± 4.1). Under physiological M5 conditions, treatment with all tested concentrations of TPM did not influence the microglia activation state (n = 18). Under pathological M30 conditions, incubation with TPM resulted in significant reduction of microglial activation (decrease of RPT) at concentrations of 5, 10, 25, and 50 µg/mL (n = 18). In parallel, the amount of RRT increased significantly and concentration-dependently under the same conditions (n = 18) (B) (TPM effects on M5 RRT: control 58% ± 6.8; 5 µg/mL 66.7% ± 3.7; 10 µg/mL 64% ± 8.7; 25 µg/mL 73.8% ± 4; 50 µg/mL 77.7% ± 2.9; TPM effects on M5 RPT: control 14.6% ± 2.4; 5 µg/mL 4.8% ± 2.2; 10 µg/mL 9.8 ± 3.3; 25 µg/mL 5.2 % ± 3.4; 50 µg/mL 4% ± 2.6; TPM effects on M30 RRT: control 10.5% ± 1; 5 µg/mL 14% ± 1.5; 10 µg/mL 19% ± 2; 25 µg/mL 32% ± 2.2; 50 µg/mL 20.5 ± 1.6; TPM effects on M30 RPT: control 57.5% ± 2.6; 5 µg/mL 47% ± 3.2; 10 µg/mL 42.3% ± 3; 25 µg/mL 32% ± 2.2; 50 µg/mL 43% ± 2.8). Comparisons between the groups were analyzed using 1-way ANOVA followed by Bonferroni post hoc comparison test. Differences were considered significant at *P < .05; **P < .01; ***P < .001; ****P ≤ .0001.

Incubation with all tested concentrations of LTG for 24 hours led to no significant alterations of the microglial morphology in M5 as well as M30 co-cultures (n = 18) (Figure 4A). Under physiological M5 conditions, treatment with all tested concentrations of TPM did not influence the microglia activation state (Figure 4B). Under pathological M30 conditions, incubation with TPM resulted in significant reduction of microglial activation at concentrations of 5, 10, 25, and 50 µg/mL (n = 18; *P < .05; **P < .01; ****P ≤ .0001) (Figure 4B). The amount of activated microglia (RPT) was decreased in a highly significant manner, from 57.47% to 32.50% (****P ≤ .0001), after 24-hour incubation at a concentration of 25 µg/mL TPM and to 43.17% (n = 18; **P < .01) at a concentration of 50 µg/mL TPM. In parallel, the amount of resting microglia (RRT) increased significantly and was concentration dependent under the same conditions. The amount of RRT microglia significantly increased from 10.58% to 20.52% (n =18; ***P < .001) after incubation with 50 µg/mL and at the maximum to 32.10% after treatment with 25 µg/mL TPM for 24 hours (n = 18; ****P < .001).

### Effects of LTG and TP on Expression of Pro-Inflammatory Cytokine TNF-α and Anti-Inflammatory Cytokine TGF-β1 in M5 and M30 Co-Cultures

**Figure 5.**
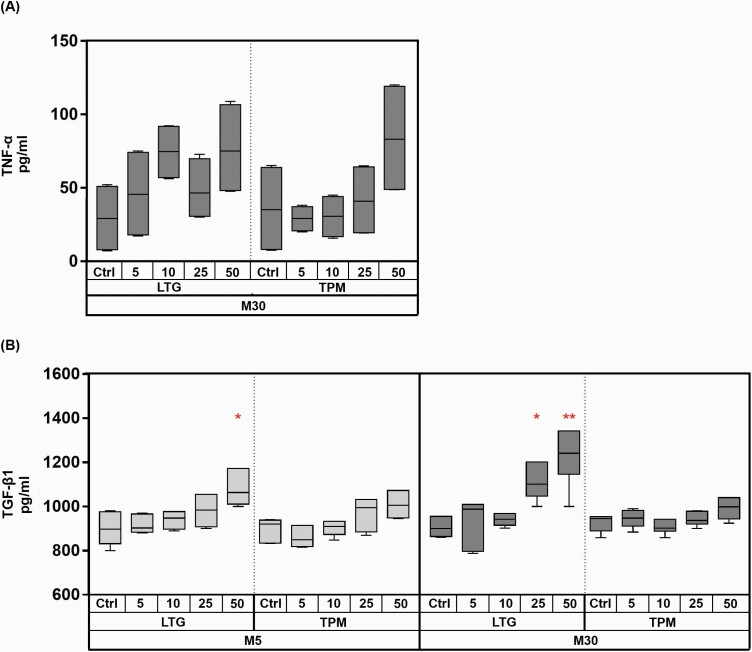
Enzyme-linked immunosorbent assay (ELISA) measured tumor necrosis factor-α (TNF-α) (n = 4) (A) and transforming growth factor-β1 (TGF-β1) (n = 3) (B) cytokine concentrations of M5 and M30 co-culture supernatants after 24-hour incubation with lamotrigine (LTG) and topiramate (TPM) (individually performed ELISAs). TNF-α levels were undetectable in the supernatants of M5 co-cultures in the controls as well as after incubation with LTG and TPM. No significant change of TNF-α cytokine levels, compared with the controls, was measured in the supernatants of M30 co-cultures after 24-hour incubation at all concentrations of LTG and TPM (A) (TNF-α after LTG incubation of M30: Ctrl 29.3 pg/mL ± 12; 5 µg/mL 45.8 pg/mL ± 15.7; 10 µg/mL 74.5 pg/mL ± 9.7; 25 µg/mL 48.9 pg/mL ± 10.6; 50 µg/mL 76.6 pg/mL ± 16.2; TNF-α after TPM incubation of M30: Ctrl 35.7 pg/mL ± 16.6; 5 µg/mL 29 pg/mL ± 4.3; 10 µg/mL 30.5 pg/mL ± 7.4; 25 µg/mL 42 pg/mL ± 12.7; 50 µg/mL 84 pg/mL ± 20). Incubation with different concentrations of TPM for 24 hours did not influence the TGF-β1 levels in M5 as well as M30 co-culture supernatants compared with controls (B) (TGF-β1 after TPM incubation of M5: Ctrl 829 pg/mL ± 30.3; 5 µg/mL 856pg/mL ± 29.8; 10 µg/mL 888pg/mL ± 24.7; 25 µg/mL 971pg/mL ± 42; 50 µg/mL 1010pg/mL ± 26.7; TGF-β1 after TPM incubation of M30: Ctrl 904pg/mL ± 27.6; 5 µg/mL 917 pg/mL ± 18; 10 µg/mL 900pg/mL ± 24; 25 µg/mL 948pg/mL ± 16.6; 50 µg/mL 987 pg/mL ± 34). Incubation with LTG, on the other hand, led to a significant increase in TGF-ß1 in both M5 and M30 co-culture supernatants (B) (TGF-β1 after LTG incubation of M5: Ctrl 904pg/mL ± 38.4; 5 µg/mL 918pg/mL ± 24; 10 µg/mL 937pg/mL ± 25.6; 25 µg/mL 978 pg/mL ± 41.6; 50 µg/mL 1098 pg/mL ± 45.5; TGF-β1 after LTG incubation of M30: ctrl 907pg/mL ± 26; 5 µg/mL 924 pg/mL ± 69; 10 µg/mL 934 pg/mL ± 19; 25 µg/mL 1136 pg/mL ± 40.2; 50 µg/mL 1256 pg/mL ± 44). Comparisons between the groups were analyzed using 1-way ANOVA followed by Bonferroni post hoc comparison test. Differences were considered significant at *P < .05, **P < .01; ***P < .001; ****P ≤ .0001. Abbreviations: Ctrl, control.

The TNF-α and TGF-β1 cytokine level in cell culture supernatants was quantified by ELISA. TNF-α levels were undetectable in the supernatants of controls as well as M5 co-cultures after incubation with LTG and TPM (n = 4; data not shown). No significant change of TNF-α cytokine levels was measured in the supernatants of M30 co-cultures after 24-hour incubation at all concentrations of LTG and TPM compared with the controls (n = 4) (Figure 5A).

Incubation with different concentrations of TPM for 24 hours did not influence the TGF-β1 levels in M5 (n = 3) as well as M30 (n = 3) co-culture supernatants compared with controls (Figure 5B). Incubation with LTG, on the other hand, resulted in a significant increase in TGF-ß1 (Figure 5B). In the physiological M5 co-culture, incubation with 50 µg/mL LTG led to a significant increase of TGF-ß1 (n = 6; *P < .05). In the pathological M30 co-culture, however, a concentration of 25 µg/mL LTG already led to a significantly increased concentration of TGF-ß1 (n = 6; *P < .05) with a maximum at 50 µg/mL LTG (n = 6; **P < .01).

### Influence of LTG and TP on Cx43 Expression in M5 and M30 Astrocyte-Microglia Co-Cultures

**Figure 6.**
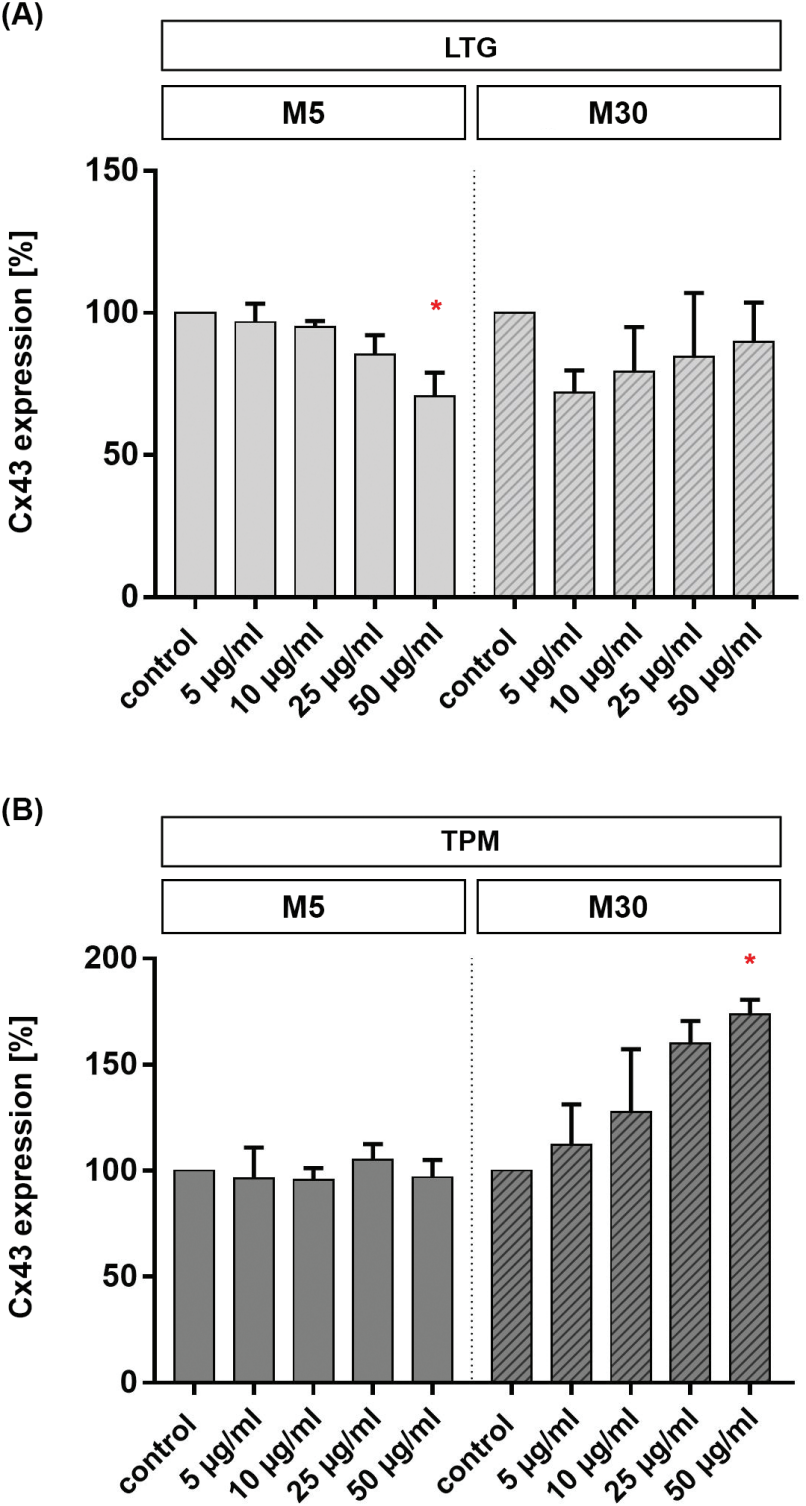
Connexin 43 (Cx43) expression after concentration-dependent incubation [5–50 µg/mL] with lamotrigine (LTG) (A) and topiramate (TPM) (B) for 24 hours in M5 and M30 co-cultures detected by western-blot analysis. The Cx43 expression in M5 co-cultures was significantly decreased after incubation with LTG for 24 hours at a concentration of 50 µg/mL (n = 3 independent experiments) (A) (Cx43 after LTG incubation of M5: control 100% ± 0; 5 µg/mL 96.8% ± 6.5; 10 µg/mL 95.2% ± 1.9; 25 µg/mL 85.4% ± 6.8; 50 µg/mL 70.8% ± 8.2). No significantly altered Cx43 protein level was measured in M30 co-cultures after concentration-dependent incubation with LTG (n = 3) (A) (Cx43 after LTG incubation of M30: control 100% ± 0; 5 µg/mL 72.8% ± 7.7; 10 µg/mL 79.4% ± 15.57; 25 µg/mL 84.5% ± 22.5; 50 µg/mL 90% ± 13.7). Incubation with different concentrations of TPM for 24 hours did not change the Cx43 expression in M5 co-cultures (B) (Cx43 after TPM incubation of M5: Ctrl 100% ± 0; 5 µg/mL 96.5% ± 14; 10 µg/mL 95.6% ± 5.6; 25 µg/mL 105% ± 7.5; 50 µg/mL 96.9% ± 8.2). Under pathological conditions in M30 co-cultures, the Cx43 expression was significantly increased after 24-hour incubation with 50 µg/mL TPM (n = 3) and weakly, but not significantly increased after incubation with 5, 10 and 25 µg/mL (B) (Cx43 after TPM incubation of M30: control 100% ± 0; 5 µg/mL 112% ± 19; 10 µg/mL 127.5% ± 29.8; 25 µg/mL 160 ± 10.5; 50 µg/mL 173.6 ± 7). Comparisons between more than 2 groups with normal distribution were analyzed using 1-way ANOVA and Kruskal-Wallis test, *P < .05; **P < .01; ***P < .001; ****P ≤ .0001.

**Figure 7.**
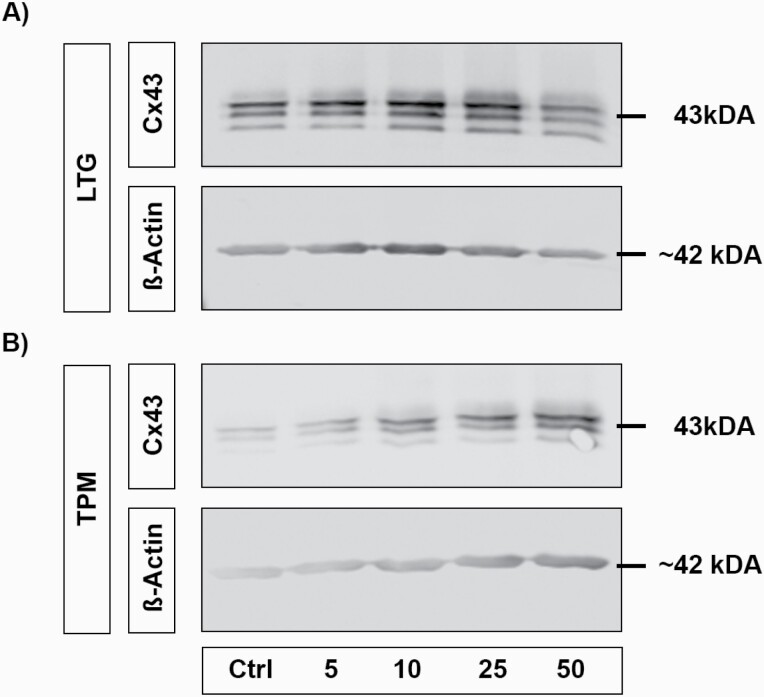
Representative western blots of connexin 43 (Cx43) in astrocyte-microglia co-cultures after treatment with lamotrigine (LTG) and topiramate (TPM) for 24 hours. Expression of all forms of Cx43 in physiological M5 co-cultures was decreased after incubation with LTG for 24 hours at a concentration of 50 µg/mL (A). Under pathological conditions in M30 co-cultures, the Cx43 expression was increased after 24-hour incubation with 50 µg/mL TPM (B). Abbreviations: Ctrl, control.

Astroglial panCx43 expression in M5 and M30 co-cultures was quantified by western blot. The total amount of expressed Cx43 content in M5 co-cultures was significantly reduced after incubation with LTG for 24 hours at a concentration of 50 µg/mL (n = 3; *P < .05) (Figures 6A and 7A). No significantly altered Cx43 protein level was measured in M30 co-cultures after concentration-dependent incubation with LTG (n = 3) (Figure 6A).

The Cx43 expression in M5 co-cultures was not changed significantly in western-blot analysis after incubation with different concentrations of TPM for 24 hours (Figure 6B). Under pathological conditions in M30 co-cultures, the Cx43 expression was significantly increased after 24-hour incubation with 50 µg/mL TPM (n = 3; *P < .05) (Figures 6B and 7B) and weakly, but not significantly, increased after incubation with 5, 10, and 25 µg/mL (Figure 6B).

## Discussion

In this study, we demonstrated a significant reduction of the glial cell viability after incubation with LTG or TPM in a concentration-dependent manner in both physiological (M5) and pathological (M30) astrocyte-microglia co-culture models. Incubation with LTG caused no significant alterations of the microglial phenotypes. Under pathological M30 conditions, a significant concentration-dependent reduction of microglial activation was detected after incubation with TPM. This correlated with increased astroglial Cx43 expression. Pro-inflammatory TNF-α cytokine levels were not affected after incubation with LTG and TPM. Incubation with higher concentrations of LTG, but not with TPM, led to a significant increase in anti-inflammatory TGF-ß1 levels in both M5 and M30 co-cultures.

### Effects of LTG and TPM on Glial Properties From Epileptological and Psychiatric Perspective

Based on previous studies, it is known that AEDs have effects on glial cells (Cardile et al., 2001; Pavone and Cardile 2003; Dambach et al., 2014). In our study, the glial cell viability was affected by LTG and TPM in a concentration-dependent manner under both physiological and pathological conditions. Another study demonstrated metabolic changes and toxic actions on astrocytes induced by LTG at higher concentrations (50 and 100 µg/mL), but not at low concentrations (1 and 10 µg/mL) (Pavone and Cardile 2003). In contrast, TPM induced stress on astrocytes at all concentrations (Pavone and Cardile 2003). These stressor effects of TPM were also confirmed on rat cortical astrocytes (Cardile et al., 2001). These findings suggest toxic effects of TPM and LTG on glia cell viability in a concentration-dependent manner. The exact mechanisms are unknown. Previous studies showed anti-apoptotic effects induced by LTG in neural precursor cells (Song et al., 2012). TPM also inhibited apoptosis by regulating the expression of apoptosis-relative genes in rat hippocampus (Su et al., 2020).

There are limited data concerning the anti-inflammatory effects of AEDs on microglial cells (Haghikia et al., 2008; Dambach et al., 2014); in particular, LTG and TPM effects on microglia are not well known (Andrzejczak et al., 2016). In the context of neuropathic pain, continuous intrathecal administration of LTG inhibited nerve ligation–induced microglial and astrocytic activation in rats (Choi et al., 2013). In our inflammatory M30 co-cultures, a significant reduction of the total number of microglia was observed after incubation with higher concentrations of LTG (25 and 50 µg/mL) and TPM (10, 25, and 50 µg/mL). Under pathological M30 conditions, TPM induced a significant concentration-dependent reduction of microglial activation. In contrast, LTG caused no significant alterations of the microglial phenotypes in M5 as well as M30 co-cultures. Our results indicate in vitro anti-inflammatory properties for TPM with regard to microglia, but not for LTG.

Pro-inflammatory TNF-α cytokine levels were not changed after incubation with LTG and TPM under all conditions. Previous findings provide strong support for the involvement of pro-inflammatory cytokines in the pathophysiology of epilepsy with elevated levels of the pro-inflammatory cytokines IL-1β, IL-6, IL-10, IL-17, IFN-α, and TNF-α (De Simoni et al., 2000; Vezzani and Granata 2005; de Vries et al., 2016). Further, elevated levels of TNF-α, IL-6 (episode of depression), and IL-23 (episode of mania) were reported in BD (Ortiz-Dominguez et al., 2007; Li et al., 2015). TNF-α was not detectable in M5 co-cultures, representing physiological conditions. Hence, activated microglia are probably the main cells, releasing TNF-α in M30 co-cultures, representing pathological inflammatory conditions. Not only TNF-α levels but also their signaling receptors on targeted cells are crucial for effects of this cytokine. Binding to TNF-α type 1 receptor was linked to cell death and hyperexcitability, whereas anti-seizure and neuroprotective effects seem to be mediated by TNF-α type 2 receptor (Weinberg et al., 2013). Previous in vitro studies using primary microglial or macrophage-like cell line cultures indicate inhibitory effects of LTG and TPM on TNF-α secretion (Andrzejczak et al., 2016; Abu-Rish et al., 2018). In our M5 and M30 astrocyte-microglia co-culture model of inflammation, this effect was not detectable. It has been demonstrated that frequently used multiple drug combinations in BD offer anti-inflammatory benefits in C8-B4 microglial cells using a combination of quetiapine and LTG (0.05 mM of each). LTG was able to reverse the pro-inflammatory effect by quetiapine concerning cytokine release (e.g., TNF-α) (Bortolasci et al., 2018). From a psychiatric point of view, it was proposed that glial inflammation leads to an increase of glutamate in the synaptic space and that this mechanism could be reduced by LTG (Haroon et al., 2017; Bortolasci et al., 2018).

Incubation with LTG, in contrast to TPM, led to a significant increase of anti-inflammatory TGF-ß1 cytokine levels in both M5 and M30 co-culture supernatants. Thus, LTG showed effects on glial cells based on cytokine release but did not affect the microglial phenotypes. Further, expression of the mainly anti-inflammatory TGF-ß1 cytokine by glial cells was associated with neuroprotective effects (Caraci et al., 2011). Following this, the neuroprotective effect of TGF-ß1 could further assist the anticonvulsant effects of LTG. On the other hand, pro-inflammatory effects of TGF-ß1 were demonstrated in primary glial cultures in which TGF-β signaling induced rapid up-regulation of IL-6 in astrocytes, but not in microglia, resulting in dysregulation of astrocyte-neuronal interactions and neuronal hyperexcitability (Levy et al., 2015). Further studies with determination of other cytokines are needed to deepen our knowledge about the exact functions of TGF-ß1 under AEDs in glial cultures. With regard to mood disorders such as BD, downregulation of TGF-β1 gene expression was described in post-mortem samples of frontal cortex tissue from patients with BD that could be relevant to the pathophysiology of the disease (Bezchlibnyk et al., 2001).

The astrocyte GJ communication was reduced in cerebral neuroinflammatory-affected tissue (Karpuk et al., 2011). In previous studies, we demonstrated positive correlation of activated microglia with reduced astroglial Cx43 expression under inflammation, suggesting that intercellular communication in the astroglial network may be modulated by the activation of microglia under in vitro conditions (Faustmann et al., 2003; Hinkerohe et al., 2005). In accordance with these findings, TPM-induced increase of Cx43 expression was associated with inhibition of microglial activation under inflammatory conditions. LTG did not change the Cx43 expression and the microglial activation in M30 co-cultures. The relevance of limited effects on Cx43 expression under physiological conditions should be viewed with caution. Cx43 expression and gap-junctional communication are also involved in the pathophysiology of mood disorders, and psychotropic drugs can influence the astrocytic network in various ways, which could be a potential novel target for mood stabilizer (Okada et al., 2020).

Previous studies have demonstrated that Cx43 resolves into multiple electrophoretic isoforms when subjected to sodium dodecyl sulfate-polyacrylamide gel electrophoresis, including a fast-migrating non-phosphorylated band termed P0 and slower-migrating phosphorylated bands termed P1, P2, and higher (Solan and Lampe 2009). In our study, we examined the modification of all forms of Cx43 (panCx43) expression under different conditions, but the phosphorylation status was not considered. Phosphorylation of Cx43 has been involved in the regulation of structure, function, localization, interaction, and channel selectivity of Cx43 as well as gap-junctional communication at several stages, for example, hemichannel oligomerization and activity and gap junction channel gating. The Cx43 phosphorylation is a dynamic process because of the short (1−5 hours) half-life of Cx43. Activation of several kinases, including protein kinase A, protein kinase C, etc., may contribute to phosphorylation (Solan and Lampe, 2009). It is an interesting issue for future studies to reveal which effects have LTG and TPM on phosphorylation of Cx43 structure and function regulating in turn gap-junctional communication and which kinases are involved under physiological and pathological conditions of our astrocyte-microglia co-culture model of inflammation.

A further limitation of our study was that only 1 method was used for the detection of Cx43 in the co-culture model; additional quantitative real-time mRNA expression analysis could be used in future projects. In addition, immunofluorescence analysis of Cx43 could be considered. Functional coupling studies of astrocytes and recording of the astrocyte membrane potential as well as investigations of further cytokines are planned for future studies. In our astrocyte-microglia co-culture model, we used the microglia/macrophage marker ED1 to visualize the microglial phenotypes/morphology with regard to activation status. However, in 2016, transmembrane protein 119 (TMEM119) was validated as a specific microglial marker for mature microglia in both mouse and human (Bennett et al., 2016; Satoh et al., 2016). There are few studies so far in which TMEM119 was also used for detection of rat microglia (Seigel et al., 2017; Zhao et al., 2020). Sialic acid–binding immunoglobulin-like lectin-H is another specific marker suitable for immunohistochemical discrimination of microglia from CNS-associated macrophages and CNS-infiltrating monocytes (Konishi et al., 2017). In contrast to TMEM119, sialic acid–binding immunoglobulin-like lectin-H is expressed by activated microglia and by microglia in immature brains in mice. Following this, it will be useful for future studies to combine ED1 immunostaining for microglia morphology with other microglia markers, for example, TMEM119 in our astrocyte-microglia co-culture model of inflammation.

In conclusion, our findings showed decreased glial cell viability after incubation with LTG and TPM in a concentration-dependent manner, indicating possible toxic effects. But both drugs were capable of reducing the extent of inflammation with regard to microglia or cytokine release in different set-ups of an in vitro astrocyte-microglia co-culture model of inflammation, contributing to better understanding mechanisms of actions of LTG and TPM in epilepsy and BD, especially possible anti-inflammatory mechanisms that lead to the therapeutic effects of both drugs. Following this, LTG and TPM could have potential positive effects on the neuroinflammatory components of the pathogenesis of epilepsy and BD. Of course, results were obtained within the in vitro co-culture model, and future investigations in animal models of epilepsy are additionally necessary for confirmation of the results. Nevertheless, the astrocyte-microglia co-culture model made possible to study the endogenous inflammatory reaction and the cytokine expression under drugs (Haghikia et al, 2008; Dambach et al., 2014).
